# 823. Urinary Bladder Matrix in Burn Care: Analysis of Use/outcomes (2021–2024) from the National Burn Repository/BCQP

**DOI:** 10.1093/jbcr/irag033.262

**Published:** 2026-04-06

**Authors:** Roselle E Crombie, Claire Witherel, Yi Arnold, Bart D Phillips, Yifei Dai, Malachy Asuku

**Affiliations:** Connecticut Burn Center/Yale New Haven Health, Westport, CT; Integra LifeSciences, Metuchen, NJ; Integra LifeSciences, Ellicott City, MD; BData Inc, Minneapolis, MN; Integra Lifesciences, Sarasota, FL; Integra Life Sciences, New Jersey, Warrington, PA

## Abstract

**Introduction:**

Burn injuries demand complex multidisciplinary management strategies that extend beyond conventional wound care to include advanced biologic and synthetic treatment options. Among these, cellular and acellular matrix-based products (CAMPs) have emerged as important tools to support tissue repair and functional recovery. Bladder matrix, an extracellular matrix–derived material, has gained traction in reconstructive surgery and wound healing, with expanding use in burn care. However, broad clinical evidence characterizing its real-world impact in this patient population is limited. Using data from the American Burn Association’s National Burn Repository (NBR) and Burn Care Quality Platform (BCQP), this study retrospectively evaluates the utilization patterns and outcomes associated with bladder matrix in burn management.

**Methods:**

A retrospective analysis of the NBR / BCQP’s resource utilization field for self-reported bladder matrix yielded a cohort of n = 325 patients treated between 2021 and 2024. Key variables extracted for analysis included patient demographics, burn etiology and severity (e.g., depth, TBSA), adjunctive therapies, anatomical locations, and clinical outcomes such as length of hospital stay (LOS) and reported complication rates.

**Results:**

Bladder matrix was most frequently applied in patients with combined second- and third-degree burns (44.1%, n = 123) and in those with isolated second-degree burns (42.3%, n = 118). Among patients with second-degree burns only, the majority (>80%, n = 103) had injuries involving <20% TBSA. In the combined second-/third-degree burn cohort, 58.6% (n = 72) presented with <20% TBSA, while 26.8% (n = 33) had burns covering 20–40% TBSA. Over 40% of all burns treated with UBM were located on the foot or leg. Common comorbidities in the overall cohort included diabetes (23.7%), tobacco use (33.7%), obesity (19.7%), and alcohol use disorder (24.4%). Reported complications were severe sepsis (3.9%), graft loss requiring reoperation (3.2%), and superficial surgical site infection (7.5%), with no cases of deep surgical site infection observed. The average length of stay was 30.1 ± 37.0 days.

**Conclusions:**

This analysis demonstrates that bladder matrix is being utilized across a broad spectrum of burn severities, most often in patients with smaller TBSA, burn injuries to the lower extremity, and patients with higher rate of comorbidities. These findings contribute real-world evidence to inform clinical decision-making and underscore the need for prospective studies to further define optimal patient selection and long-term outcomes associated with UBM use in burn care.

**Applicability of Research to Practice:**

As technology has become commonplace use in burns, impact of the technology and search for more economic alternatives need to be evaluated.

**Funding for the study:**

Acquisition of visibility into the database was done through an educational grant.

